# Comparison of biophysical properties of α1β2 and α3β2 GABA_A_ receptors in whole-cell patch-clamp electrophysiological recordings

**DOI:** 10.1371/journal.pone.0234080

**Published:** 2020-06-01

**Authors:** Emma Rie Olander, Dieter Janzen, Carmen Villmann, Anders A. Jensen

**Affiliations:** 1 Institute for Clinical Neurobiology, Julius-Maximilians-Universität Würzburg, Würzburg, Germany; 2 Department of Drug Design and Pharmacology, Faculty of Health and Medical Sciences, University of Copenhagen, Copenhagen, Denmark; Indiana University School of Medicine, UNITED STATES

## Abstract

In the present study we have characterized the biophysical properties of wild-type (WT) α1β2 and α3β2 GABA_A_ receptors and probed the molecular basis for the observed differences. The activation and desensitization behavior and the residual currents of the receptors expressed in HEK293 cells were determined in whole-cell patch clamp recordings. Kinetic parameters of α1β2 and α3β2 activation differed significantly, with α1β2 and α3β2 exhibiting rise times (10–90%) of 24 ± 2 ms and 51 ± 7 ms, respectively. In contrast, the two receptors exhibited largely comparable desensitization behavior with decay currents that could be fitted to exponential functions with two or three components. Most notably, the two receptor compositions displayed different degrees of desentization, with the residual currents of α1β2 and α3β2 constituting 34 ± 2% and 21 ± 2% of the peak current, respectively. The respective contributions of the extracellular domains and the transmembrane/intracellular domains of the α-subunit to these physiological profiles were next assessed in recordings from cells expressing αβ2 receptors comprising chimeric α-subunits. The rise times displayed by α1^ECD^/α3^TMD^β2 and α3^ECD^/α1^TMD^β2 receptors were intermediate to those of WT α1β2 and WT α3β2, and the distribution of the different components of the current decays exhibited by the two chimeric receptors followed the same pattern as the two WT receptors. The residual current exhibited by α1^ECD^/α3^TMD^β2 (23 ± 3%) was similar to that of α3β2 but significantly different from that of α1β2, whereas the residual current displayed by α3^ECD^/α1^TMD^β2 (27 ± 2%) was intermediate to and did not differ significantly from either of the WT receptors. This points to molecular differences in the transmembrane/intracellular domains of the α-subunit as the main determinants of the observed differences in receptor physiology between α1β2 and α3β2 receptors.

## Introduction

γ-Aminobutyric acid (GABA) is the major inhibitory neurotransmitter in the CNS and also found in several peripheral tissues, where it is involved in and regulates a vast number of physiological processes. Most of GABAergic transmission is mediated via GABA_A_ receptors (GABA_A_Rs), a family of pentameric anion-selective GABA-gated channels [1, 2]. Because of their roles as key regulators of neuronal excitability in the CNS, GABA_A_Rs are being pursued as putative therapeutic targets for a wide range of neurological, cognitive and psychiatric disorders, and the receptors are currently targeted by clinical drugs for sleep disorders, anxiety and epilepsy and by various anaesthetics [2–5].

The heterogeneity of the GABA_A_R family arises from the existence of 19 different subunits (α_1_-α_6_, β_1_-β_3_, γ_1_-γ_3_, δ, ε, π, θ, ρ_1_-ρ_3_) that assemble into at least 30 different receptor subtypes *in vivo* [1, 2]. While the majority of GABA_A_Rs in the CNS are assembled by two α subunits, two β subunits and a γ subunit, the functions mediated by these ternary αβγ receptors are supplemented by important contributions from other GABA_A_Rs, such as αβδ receptors, binary αβ receptors and homomeric/pseudo-homomeric ρ receptors [1, 2]. Thus, the overall orchestration and fine-tuning of GABAergic neurotransmission is very much rooted in the differential regional expression patterns and neuronal distribution of the different GABA_A_R subunits and the resulting distinct signaling characteristics exhibited by the various assembled subtypes.

Signaling through the pentameric GABA_A_R complex is triggered by agonist binding to the orthosteric sites located in the extracellular domain (ECD), which causes the opening of the ion channel in the transmembrane domain (TMD) of the receptor and enables the flux of Cl^-^ and other anions through the channel [2, 6–8]. Following this activation, the receptor will eventually undergo a transition into a desensitized state, in which the ion channel collapses into a closed state despite the agonist still being bound to the receptor. This shapes the current through the receptors and consequently the membrane potential of the cell, which in turn affects the firing of the neuron and thereby the activity of the nervous system [9, 10]. The agonist-mediated transitions between resting, active and desensitized GABA_A_R conformations are determined by the energy barriers between the respective states, and the different kinetic properties exhibited by an agonist at different receptor subtypes are rooted in differences in these energy barriers between the different GABA_A_R assemblies [2, 11]. αβγ GABA_A_Rs exhibit considerably faster activation kinetics than αβ and αβδ receptors, whereas αβδ receptors desensitize faster than both αβγ and αβ receptors [12]. The identity of the β-subunit has been shown to impact the kinetic properties exhibited by recombinant α5βγ2 receptors in HEK293 cells substantially in recordings from a neuron-HEK293 cell co-culture system [13], and the different α-subunits are also known to confer different biophysical properties to the αβγ receptors. For example, α3βγ2 receptors (α3β2γ2S and α3β3γ2L) have been reported to exhibit slower time courses of activation, deactivation and desensitization than their corresponding α1βγ2 receptors (α1β2 γ 2S and α1β3 γ 2L) [14, 15], even though these differences are more subtle than the overall differences observed between the αβγ, αβ, αβδ and ρ receptors [12, 16].

In the present study, we have investigated the biophysical properties of α1β2 and α3β2 GABA_A_Rs expressed in HEK293 cells by whole-cell patch-clamp recordings and studied the molecular basis for the observed differences in activation and desensitization properties exhibited by the two wild-type (WT) receptors. Analogously to previous studies of α1- and α3-containing receptors using excised patch clamp electrophysiology [14, 15], we find that different components of α1β2 and α3β2 GABA_A_R kinetics also differ in this set-up, with α3β2 exhibiting significantly slower rise time during its activation and bigger residual current following desensitization than α1β2. Finally, the transmembrane and/or intercellular domains of the α-subunit are shown to comprise the key molecular determinants underlying the observed differences in channel activation and desensitization behavior.

## Materials and methods

### Materials

GABA and chemicals used for buffers were obtained from Sigma-Aldrich (St. Louis, MO). Diazepam was a kind gift from Dr. Henrik S. Jensen (H. Lundbeck A/S). The cDNAs encoding for the human GABA_A_R subunits α1, α3, β2 and γ2S were kind gifts from Dr. Paul J. Whiting. The subcloning of the these cDNAs into the pCDNA3.1 vector and the construction of the cDNAs encoding for the chimeric α1^ECD^/α3^TMD^ and α3^ECD^/α1^TMD^ subunits have been described previously [17, 18]. An alignment of the amino acid sequences of the α1 and α3 TMDs (including the fusion points in the two chimeras) is given in S1 Fig.

### Cell culture and transfections

Human Embryonic Kidney 293 (HEK293) cells (ATCC, Manassas, VA) were cultured in minimum essential medium (Life Technologies, Darmstadt, Germany) supplemented with 10% fetal bovine serum, 1 mM sodium pyruvate, and 50 U/ml penicillin/streptomycin (all supplements from Thermo Fisher Scientific, Waltham, MA) at 37°C in a humidified atmosphere with 5% CO_2_. Cells were passaged every 4 days and used for experiments when they were 70–90% confluent.

Cells were transiently transfected using the calcium-phosphate precipitation technique. For the αβγ GABA_A_Rs, 0.3 μg α-subunit cDNA (α1-pcDNA3.1 or α3-pcDNA3.1), 0.3 μg β2-pcDNA3.1, 1.5 μg γ 2S-pcDNA3.1 and 0.5 μg pEGFP-N1 was used for transfection of a 9.6 cm^2^ tissue culture dish of HEK293 cells. For the αβ GABA_A_Rs, 0.5 μg α-subunit cDNA (α1-pcDNA3.1, α3-pcDNA3.1, α1^ECD^/α3^TMD^-pcDNA3.1 or α3^ECD^/α1^TMD^-pcDNA3.1), 0.5 μg β2-pcDNA3.1 and 0.5 μg pEGFP-N1 was used for transfection of a 9.6 cm^2^ tissue culture dish of HEK293 cells. GFP was included to identify transfected cells under fluorescent light during the recordings. The cDNA was mixed with 0.1x TE-buffer (pH 8.0) (AppliChem GmbH, Darmstadt, Germany), 2.5 M CaCl_2_, and 2x HBS Puffer (12 mM D-glucose, 10 mM KCl, 280 mM NaCl, 1.5 mM Na_2_HPO_4_, 50 mM HEPES, pH 6.95) and incubated for 20 min before application to the cells. Cells were incubated with the solution for 6 hours, after which the medium was removed and replaced with fresh cell medium. Electrophysiological recordings were performed 24–48 h after transfection.

### Electrophysiological recordings

Glass pipettes were pulled on the day of the experiment on a Flaming-Brown P97 micropipette puller (Sutter Instrument, Novato, CA) from borosilicate capillaries to a resistance of 4–10 MOhm. Pipettes were filled with intracellular solution (120 mM CsCl, 20 mM N(Et)_4_Cl, 1 mM CaCl_2_, 2 mM MgCl_2_, 11 mM EGTA, 10 mM HEPES, pH 7.2). The whole-cell patch clamp technique was used to record currents from cells. After obtaining a gigaohm seal cells were opened by suction and voltage clamped to -60 mV. Current responses were measured at room temperature (21–23°C) at the holding potential of −60 mV. Solutions were applied using an Octaflow II system (ALA Scientific Instruments, Farmingdale, NY), where cells were bathed in a laminar flow of buffer. In a previous study using the same application system the time resolution for solution exchange and re-equilibration in whole-cell recordings from HEK293 cells has been determined to be about 100 ms [19]. The Octaflow resolution is 10–30 ms, while the time for the whole cell to be surrounded by agonist may be a bit slower, and the solution exchange thus is estimated to 100 ms. Cells were continually perfused with extracellular solution (137 mM NaCl, 5.4 mM KCl, 1.8 mM CaCl_2_, 1.0 mM MgCl_2_, 10 mM HEPES, pH 7.4). To measure GABA-evoked currents, 30 mM GABA was applied to the cells for a period of 28 s. Currents were amplified and digitized using an EPC-10 amplifier (HEKA, Lambrecht, Germany) and the software Patchmaster (HEKA, Lambrecht, Germany). Currents were filtered at 2.9 kHz and digitized at 10–13.3 kHz.

### Data analysis

The patch clamp data was analysed by OriginPro 2017 (OriginLab Corporation, Northampton, MA). The peak current was measured from baseline to peak. Rise time was calculated as the time between the 10% current level and the 90% current level of the rising phase of the current. The decaying phase of the current was fitted with an exponential function containing two or three exponential components and a constant representing a steady state corresponding to the residual current: y(t) = A_1_exp^(-t/τ1)^ + A_2_exp^(-t/τ2)^ + A_3_exp^(-t/τ3)^ + S, where τ_1_, τ_2_, and τ_3_ are the time constants, A_1_, A_2_, and A_3_ the fractions of each component, and S the steady state current. Weighed tau was calculated as τ_W_ = A_1_τ_1_+A_2_τ_2_+A_3_τ_3_, where τ_1_, τ_2_, and τ_3_ are the fitted time constants and A_1_, A_2_, and A_3_ the corresponding fitted fractions. Residual current was calculated as the percentage of peak current determined 28 s after the peak current: [residual current/(peak current–baseline current)].

### Statistical analyses

Statistical analyses were performed to determine significant differences between properties of different receptor subtypes. For comparison of two subtypes students t-test was performed. For comparison of more than two groups one-way ANOVA was performed followed by Tukey’s multiple comparisons test. Brown-Forsythe and Welch ANOVA was used for unequal standard deviations where appropriate followed by Games-Howell’s multiple comparisons test. F-tests were used to determine if increasing the number of exponential components significantly improved the fit of the data. Statistical analyses were performed by GraphPad Prism 8 (GraphPad Software, Inc., San Diego, CA).

## Results

### Expression and characterization of the biophysical properties of binary αβ receptors

To characterize and compare kinetic properties of α1- and α3-containing GABA_A_Rs, we expressed the receptors in HEK293 cells and applied the whole-cell patch clamp technique. Initially, cells were transfected with the three subunits encoding for the α1β2γ2S and α3β2γ2S receptors. The presence of the γ2S-subunit in cell surface expressed receptors was assessed by quantifying the modulation mediated by a saturating concentration of diazepam (1 μM), a αβγ-selective positive allosteric modulator, of the currents evoked through the receptors by EC_20_ (EC_10_-EC_30_) GABA. The degree of diazepam-mediated modulation of GABA-evoked responses varied considerably between different recording days (and different transfections), even though the cells were cultured and transfected under identical conditions (S2 Fig). This could be a reflection of variation in the degree of incorporation of the γ2S-subunit into the cell surface-expressed receptors. Because of these inconsistencies and to ensure that the recordings from the HEK293 cells were done from a homogenous receptor population, we decided to study the kinetic properties of the binary α1β2 and α3β2 receptors.

### Characterization of the kinetic properties of WT α1β2 and α3β2 receptors

**Fig 1:**
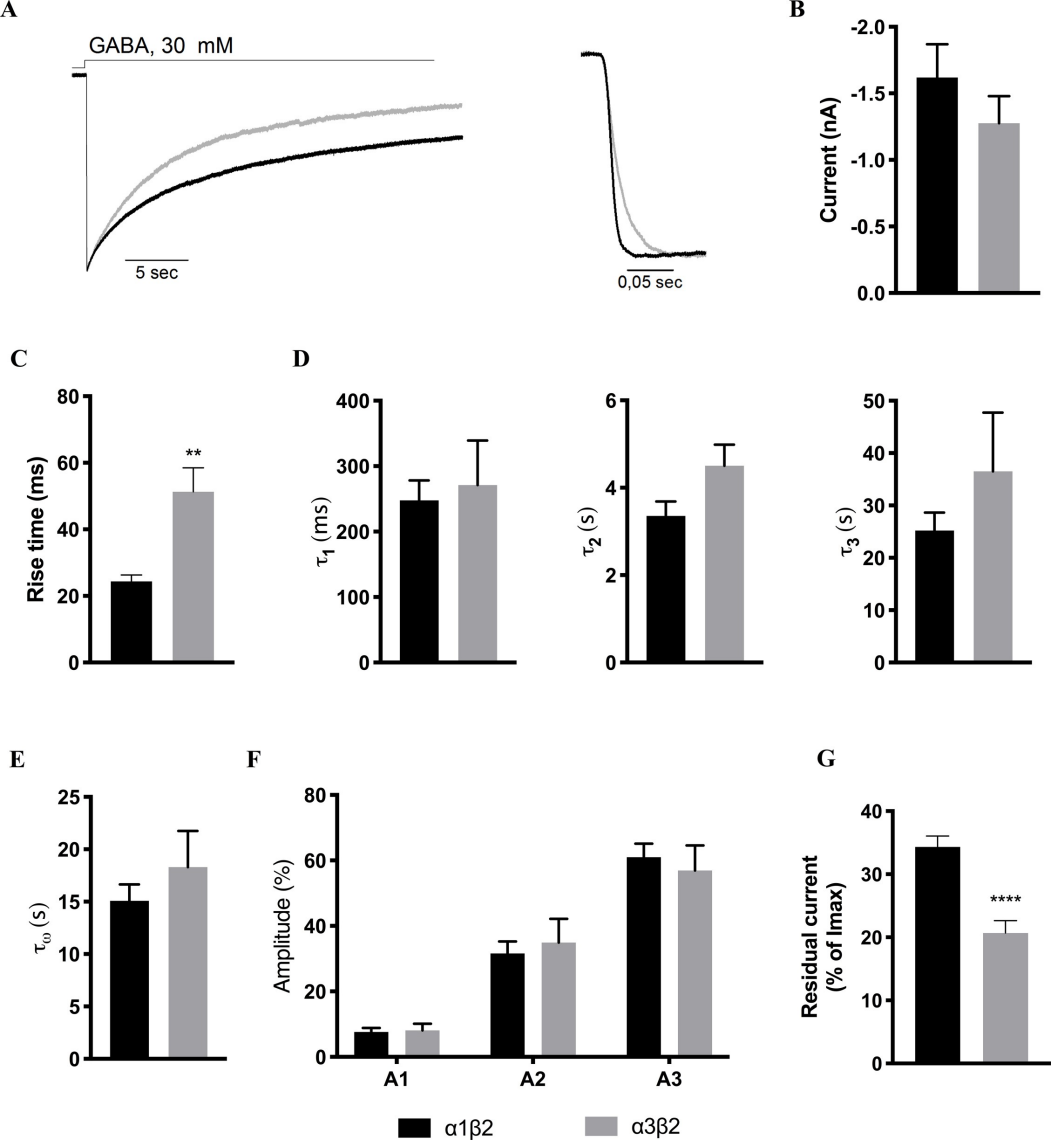
Kinetic properties exhibited by WT α1β2 and α3β2 receptors. A. Representative current traces for wild-type α1β2 (black) and α3β2 (grey) receptors (*left*) and the activation phases of α1β2 (black) and α3β2 (grey) from representative current traces (*right*) B. Averaged peak currents for α1β2 (black) and α3β2 (grey). The n-values are given in Table 1. C-G. Averaged values (mean ± S.E.M.) for various kinetic parameters for α1β2 (black) and α3β2 (grey) are given. Statistical differences are indicated with asterisks (Welch’s ANOVA, * P<0.05). The averaged data and n-values are given in Table 1. *C*. *Activation*. Averaged rise times (10–90%) of the activation phase exhibited by α1β2 and α3β2 receptors. *D-F*. *Decay*. τ_1_, τ_2_, and τ_3_ are given in D, τ_W_ values are given in E and the current fractions A_1_, A_2_ and A_3_ in F. *G*. *Residual currents*. The averaged residual currents for the two WT receptors are given.

The kinetic properties of activation and desensitization of the currents evoked by application of a high concentration of GABA (30 mM) at α1β2- and α3β2-expressing HEK293 cells were next investigated (Fig 1A). The average current levels recorded from cells transfected with WT α1β2 and WT α3β2 were similar, 1.6 ± 1.0 nA (n = 17) and 1.3 ± 0.2 nA (n = 15), respectively, thus allowing for direct comparisons of their respective kinetic properties (Fig 1B).

#### Receptor activation

As can be seen from the activation phases in the representative current traces for α1β2 and α3β2 (Fig 1A, *right*), the activation kinetics of the two receptors differed significantly, with the average rise times (10–90%) of 24 ± 2 ms for α1β2 (n = 17) and 51 ± 7 ms for α3β2 (n = 13, two of the 15 recordings were excluded from the rise time calculations due to noise spikes on the curve that precluded analysis) (Welch’s t-test, P = 0.0025) (Fig 1C, Table 1).

**Table 1:** Biophysical properties exhibited by WT α1β2, WT α3β2, α1^ECD^/α3^TMD^β2 and α3^ECD^/α1^TMD^β2 GABA_A_Rs expressed in HEK293 cells in whole cell patch clamp recordings.

Receptor	Rise time [ms]	τ_1_ [ms]	τ_2_ [s]	τ_3_ [s]	τ_W_ [s]	A_1_ [%]	A_2_ [%]	A_3_ [%]	*Residual Current* [%]
α1β2	24 ± 2 *(17)*	248 ± 30 *(12)*	3.4 ± 0.3 *(12)*	25.2 ± 3.4 *(12)*	15.5 ± 1.7 *(17)*	7.5 ± 1.2 *(12)*	32 ± 4, *(12)*	61 ± 4 *(12)*	34 ± 2 *(17)*
α3β2	51 ± 7 *(13)*	271 ± 68 *(9)*	4.5 ± 0.5 *(9)*	36.5 ± 11.2 *(9)*	18.3 ± 3.4 *(15)*	8.1 ± 2.0 *(9)*	35 ± 7, *(9)*	57 ± 8 *(9)*	21 ± 2 *(15)*
α1^ECD^/α3^TMD^β2	28 ± 5 *(12)*	108 ± 14 *(3)*	2.9 ± 1.8 *(3)*	11.4 ± 3.2 *(3)*	9.6 ± 3.6 *(12)*	9.9 ± 4.4 *(3)*	24 ± 17, *(3)*	66 ± 15 *(3)*	23 ± 3 *(12)*
α3^ECD^/α1^TMD^β2	36 ± 9 *(13)*	311 ± 84 *(10)*	4.0 ± 1.8 *(10)*	26.3 ± 8.1 *(10)*	15.3 ± 3.9 *(14)*	9.7 ± 1.7 *(10)*	27 ± 3, *(10)*	63 ± 13 *(10)*	27 ± 2 *(14)*

The number of experiments (n) is given in italics in parenthesis for each value.

#### Receptor decay

For current sweeps that both could be fitted well with both a two-phase exponential function and a three-phase exponential function, an F-test was performed to determine the best fit. For the α1β2 receptor, 12 of the 17 sweeps were fitted with a three-phase exponential function with a fast, an intermediate, and a slow phase, whereas four sweeps were best fitted with an exponential function with two phases. For the α3β2 receptor, 9 of 15 sweeps were best fitted with a three-phase exponential function, whereas six sweeps were best fitted with two-phase exponential functions.

To enable direct comparisons, only sweeps fitted with three-phase exponential functions were included for the comparison of τ_1_, τ_2_ and τ_3_ values and the corresponding current fractions (A_1_, A_2_, A_3_) between the two receptors. All recorded sweeps were included in the calculation of the weighed τ-value (τ_W_) between the receptors. As outlined in Fig 1D and Table 1, the three mean τ-values obtained for the two receptors were very similar, with τ_1_, τ_2_ and τ_3_ being 248 ± 30 ms, 3.4 ± 0.3 s and 25.2 ± 3.4 s for α1β2 and 271 ± 68 ms, 4.5 ± 0.5 s and 36.5 ± 11.2 s for α3β2. The weighed τ-value did not differ significantly between the receptors either, τ_W_ being 15.47 ± 1.72 s and 18.31 ± 3.43 s for α1β2 and α3β2, respectively (Fig 1E). The distribution of current decay between the fast, intermediate and slow phases was also similar for α1β2 and α3β2 (see A_1,_ A_2_ and A_3_ values in Fig 1F and Table 1). For both receptor types, the fast phase only constituted a small fraction of the total current (7.5 ± 1.2% for α1β2, 8.1 ± 2.0% for α3β2), the intermediate phase was considerably larger (32 ± 4% for α1β2, 35 ± 7% for α3β2), and the slow phase fitted the largest current fraction for both receptors (61 ± 4% for α1β2, 57 ± 8% for α3β2) (Fig 1F; Table 1).

#### Residual current

In the analysis of the residual currents (determined 28 s after the peak), α1β2 and α3β2 exhibited averaged residual currents of 34 ± 2% (n = 17) and 21 ± 2% (n = 15) after 28 s GABA application, respectively (Fig 1G; Table 1). Thus, the α3β2 receptor was found to desensitize to a significantly larger extent than the α1β2 receptor within this timeframe (P<0.0001, unpaired t-test).

### Characterization of the biophysical properties of the α1ECD/α3TMDβ2 and α3ECD/α1TMDβ2 receptors

**Fig 2:**
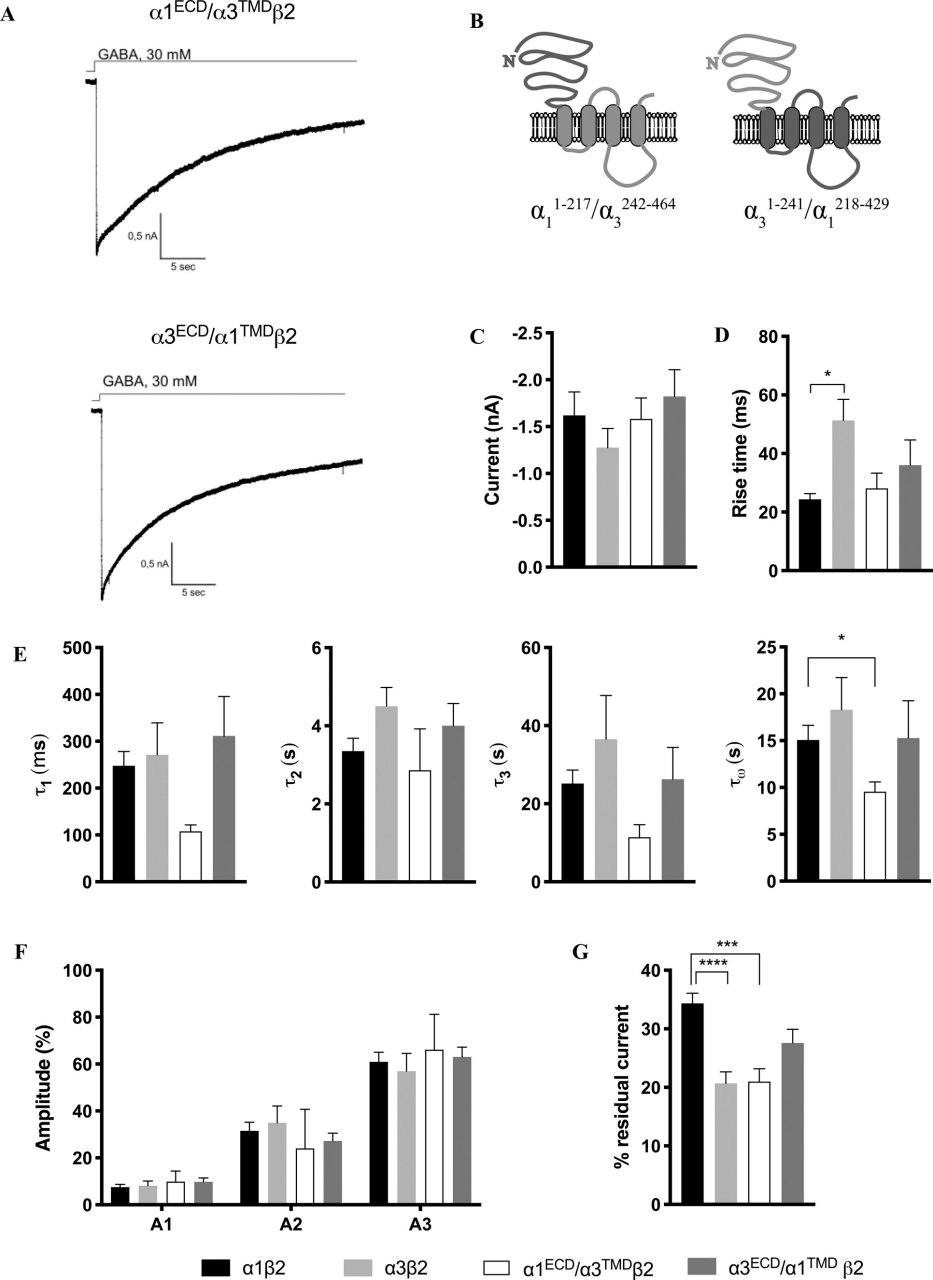
Kinetic properties exhibited by the α1^ECD^/α3^TMD^β2 and α3^ECD^/α1^TMD^β2 receptors. A. Representative current traces for α1^ECD^/α3^TMD^β2 and α3^ECD^/α1^TMD^β2. B. Topologies of the chimeric α1^ECD^/α3^TMD^ and α3^ECD^/α1^TMD^ subunits. C. Averaged peak currents for α1^ECD^/α3^TMD^β2 (white) and α3^ECD^/α1^TMD^β2 (dark grey) together with the corresponding data for WT α1β2 (black) and α3β2 (light grey) receptors (from Fig 1). n-values are given in Table 1. D-G. Averaged values (mean ± S.E.M.) for various kinetic parameters for α1^ECD^/α3^TMD^β2 (white) and α3^ECD^/α1^TMD^β2 (dark grey) are given together with the corresponding data for WT α1β2 (black) and α3β2 (light grey) receptors (from Fig 1) for comparison. Statistical differences are indicated with asterisks (Welch’s ANOVA, * P<0.05). The averaged data and n-values are given in Table 1. *D*. *Activation*. The rise times for the four receptors are given. *E and F*. *Decay*. τ_1_, τ_2_, τ_3_ and τ_W_ values (E) and current fractions A_1_, A_2_ and A_3_ (F) are given. *G*. *Residual currents*. The residual currents for the four receptors are given.

In an attempt to elucidate the molecular basis for the different kinetic properties exhibited by the α1β2 and α3β2 receptors and to identify the receptor domain comprising the molecular determinants of these differences, we next studied the signaling properties of two binary receptors assembled from a chimeric α subunit and the WT β2 subunit. The α1^ECD^/α3^TMD^ chimera comprises the extracellular *N*-terminal domain of α1 and the transmembrane and intracellular domains of α3, and conversely the α3^ECD^/α1^TMD^ chimera comprises the *N*-terminal domain of α3 and the transmembrane and intracellular domains of α1 (Fig 2B). The chimeric subunits were co-expressed together with β2 in HEK293 cells, their signaling properties were characterized by whole-cell patch clamp recordings (a total of 10 and 14 recordings were performed for α1^ECD^/α3^TMD^β2 and α3^ECD^/α1^TMD^β2, respectively), and the data were analysed in the same way as for the WT α1β2 and α3β2 receptors (Fig 2; Table 1). The average current levels recorded from cells transfected with α1^ECD^/α3^TMD^β2 (1.6 ± 0.2 nA, n = 10) and α3^ECD^/α1^TMD^β2 (1.8 ± 0.3 nA, n = 14) were comparable to those displayed by WT α1β2 and WT α3β2, thus allowing for direct comparisons of their respective kinetic properties.

#### Receptor activation

As can be seen from Figs 1C and 2D, the activation kinetics of both α1^ECD^/α3^TMD^β2 and α3^ECD^/α1^TMD^β2 were intermediate to those of WT α1β2 and WT α3β2. The average rise times exhibited by α1^ECD^/α3^TMD^β2 and α3^ECD^/α1^TMD^β2 were 28 ± 5 ms and 36 ± 9 ms, respectively, and thus they did not differ significantly from each other or from the average rise times exhibited by the two WT receptors.

#### Receptor decay

Analogously to the fitting of the data for the two WT receptors, the decaying phase of the current was fitted with functions with two or three exponential components. For α1^ECD^/α3^TMD^β2, only three of 12 sweeps were fitted with a three-phase exponential function, whereas the remaining sweeps were best fitted with a two-phase exponential function. For α3^ECD^/α1^TMD^β2, 10 of 14 sweeps were fitted with a three-phase exponential function, and two were fitted with a two-phase exponential function. The τ_1_ and τ_3_ values displayed by α1^ECD^/α3^TMD^β2 were smaller than the values obtained for the three other receptors. These differences were not statistically significant, which in part may be attributable to the fact that only a few of the current sweeps for this receptor could be fitted with a three-exponential phases function (n = 3). The τ_W_ for α1^ECD^/α3^TMD^β2 was also smaller than the τ_W_ for the other three receptors and significantly different from the τ_W_ for WT α1β2 (P = 0.0133, Welch’s ANOVA test). The distribution of the current decay between the fast, intermediate, and slow phase for α1^ECD^/α3^TMD^β2 and α3^ECD^/α1^TMD^β2 followed the same pattern as observed for the WT α1β2 and α3β2 receptors with the fast phase contributing to the smallest fraction of the current and the slow phase to the largest (see A_1,_ A_2_ and A_3_ values in Fig 2F and Table 1).

#### Residual current

Analysis of the residual currents displayed by the two chimeric receptors found that α1^ECD^/α3^TMD^β2 and α3^ECD^/α1^TMD^β2 exhibited residual currents of 23 ± 3% (n = 10) and 27 ± 2% (n = 14) after 28 s GABA application, respectively (Fig 2G, Table 1). Thus, the average residual current of α1^ECD^/α3^TMD^β2 was comparable to that of WT α3β2 (21 ± 2% (n = 15)), whereas it differed significantly from that of WT α1β2 (34 ± 2% (n = 17)) (Adjusted P-value 0.0012, one-way ANOVA). The residual current exhibited by α3^ECD^/α1^TMD^β2 was intermediate to those of α1β2 and α3β2, and it was not significantly different from the values identified for the two WT receptors.

## Discussion

In the present study, we have studied activation and desensitization parameters of the human α1β2 and α3β2 GABA_A_Rs and probed the molecular basis for the observed differences between these by use of whole-cell patch-clamp electrophysiology. The activation of the α1β2 receptor was found to be faster than α3β2, and none of the decay parameters determined for the receptors differed. However, the degree of their desensitization did, with the residual current for the α3β2 receptor being significantly larger than that of α1β2. Finally, delineation of the biophysical properties exhibited by chimeric α1^ECD^/α3^TMD^β2 and α3^ECD^/α1^TMD^β2 receptors demonstrated the major molecular determinants of the observed differences in activation and desensitization to reside within the transmembrane and/or intracellular domains of the α-subunit.

Previous studies applying excised-patch clamp electrophysiology have found that the desensitization kinetics of α1-containing receptors are faster than the desensitization kinetics of α3-containing receptors. Our study shows that in the whole-cell patch-clamp electrophysiology mode such differences are not observed. An important consideration is to which extent the findings for the α1β2 and α3β2 receptors reported here can be compared to those displayed by α1- and α3-containing GABA_A_Rs in previous studies applying excised-patch recordings [14, 15] or whether the experimental set-ups in our and these studies are too different for such comparisons. In light of these experimental differences, it is not surprising that the absolute values of the kinetic parameters, the relative distribution of the two or three components in the current decay as well as the degree of desensitization exhibited by α1β2 and α3β2 in the present study differed substantially from those in the previous studies. Nevertheless, we propose that the previous recordings contribute to our understanding of which biophysical properties of the two receptors recorded using the whole-cell patch-clamp method.

### Receptor activation of α1β2 and α3β2

The activation of α1β2 were found to be twice as fast as that of α3β2 (Fig 1C), which qualitatively is in good agreement with the previous findings in the previous studies of α1- and α3-containing receptors. In the present study, the averaged 10–90% rise times exhibited by α1β2 (24 ms) and α3β2 (51 ms) in the whole-cell recordings are on a different time scale compared to those previously determined for α1β3 γ 2L (0.60 ms) and α3β3 γ 2L (1.8 ms) [15], for α1β2 γ 2 (0.29 ms) and α3β2 γ 2 (0.41 ms) [14], and for α1β3 and α1β3 γ 2L receptors [12, 20] in excised-patch recordings. These substantial differences are unlikely to arise from the different receptor compositions studied, as evidenced by a rise time as low as of 1.7 ms reported for α1β3 in one of these studies [12], but instead has to be ascribed to the different methodologies used. Indeed, Bianchi and Macdonald have previously reported dramatically different activation kinetics for α1β3 γ 2L depending on the method used, ranging from rise times of ~100 ms using a conventional whole cell patch clamp application system, over rise times of ~7 ms achieved with a faster application system, to rise times <1 ms using excised-patch recordings [20]. These results clearly show that the application system and recording method influence the current rise time, and that the rise time in whole cell recordings are not reflective of the activation time of individual receptors. Additionally, the slower application time can result in smaller peak currents, as not all receptors on the cell are activated simultaneously. The receptors in the cell membrane located closest to the application pipette will be activated and start to desensitize before receptors further away from the pipette are activated, which means that the evoked current in the cell arises from a mix of desensitized and active receptors. This receptor mixture reduces the peak current measured compared to the peak that should have been achievable, if all receptors were activated simultaneously. However, our data also show that despite the slower application time in our system, the difference in rise time between the α1β2 and α3β2 receptor is still observed, and the faster activation of the α1β2 receptor compared to the α3β2 receptor is reflected in the whole cell patch clamp recordings.

### Receptor desensitization of α1β2 and α3β2

The decay phase of both α1β2 and α3β2 was mostly fitted well with an exponential function containing three phases: a fast phase (~250 ms), an intermediate phase (~4 s), and a slow phase (~30 s) (Fig 1). The τ-values for the fast, intermediate, and slow phases as well as τ_W_ were similar between α1β2 and α3β2, and thus no difference was observed for the desensitization kinetics of the two receptors. One study applying exised-patch recordings on α1β2 γ 2 and α3β2 γ 2 GABA_A_Rs expressed in HEK293 or HEK293T cells have observed slower desensitization kinetics of α1β2γ2 compared to α3β2γ2 [14], whereas another study comparing α3β3γ2L to α1β3γ2L did not observe any significant differences between the two receptors [15]. In both studies, however, the relative distribution of the three exponential phases (fast, intermediate, slow) in the fitted data in these studies also differ from those in our data, with the fast component (τ_1_) contributing with the largest or a major fraction of current (A_1_) in the exised-patch recordings [14, 15] and the slow component (τ_3_) contributing with the largest fraction of current (A_3_) in our recordings. In the study by Barberis et al. [14] the fast component accounted for more than 50% of the current for α1β2 γ 2 and only 20% for α3β2 γ 2. Since it is not possible to resolve a desensitization time component faster than the recorded rise time of the peak current, we would not expect to be able to resolve the fastest time constant in our recording set-up. However, it should be possible to resolve the intermediate time constants, which were found to be ~250 ms in the exiced-patch recording studies [14, 15]. This points to that other factors than the application speed may contribute to the difference between this and the exised-patch recording studies.

It is unlikely that the slower desensitization in this study is a reflection of the αβ receptors compared to the αβ γ receptors investigated in previous studies [14, 15], as αβ and αβ γ receptors have been shown to desensitize with similar time courses [12]. Instead, the difference in the sizes of the cell between whole cell patch clamp and excised patch clamp recordings could potentially also contribute to the differences in receptor desensitization. Whereas currents are recorded from a relatively large cells in whole cell patch clamp, in the excised patch recordings the small piece of cell membrane forms a very small cell around the pipette tip. It has previously been shown that intracellular Cl^-^ concentrations greatly affect the time course of current decay [21]. Activation of GABA_A_Rs on the cell surface leads to a fast rise in intracellular Cl^-^. As a consequence the driving force for Cl^-^ changes simultaneously, leading to a rapid current decay. The decrease in driving force is much more pronounced in a small cell compared to a large cell, and it is possible that the rapid decay kinetics reported for GABA_A_ receptors in excised-patch clamp studies reflect a rapid change in Cl^-^ rather than fast desensitization kinetics. Our data show that desensitization kinetics are similar between the α1β2 and α3β2 receptors in the whole cell patch clamp setup.

### Residual currents for α1β2 and α3β2

Both receptors exhibited a high percentage residual current after 28 s of GABA application, 34% and 21% for α1β2 and α3β2, respectively. This apparent larger extent of desensitization of α3β2 than of α1β2 has to our knowledge not previously been reported, and it seems to contrast the largely comparable time course of desensitization of the two receptors (Fig 1D–1F). As mentioned above, the faster activation kinetics exhibited by α1β2 compared to α3β2 may in part arise from the slower rate of agonist application in the set-up in this study (Fig 1C). The residual current is determined relative to the peak current, and a smaller peak current will thus cause the residual current to be relatively larger. Since α1β2 activates faster than α3β2, it is possible that the measured peak current for the α1β2 receptor is smaller compared to the”theoretical maximum possible peak current” which thereby would result in the apparently larger residual current. On the other hand, the slower activation time course of α3β2 could also be reflective of this receptor reactivating more slowly during the desensitization phase of the currents than α1β2, which could contribute to its lower degree of desensitization and higher residual current.

In our investigation of which domains of the α1/3β2 receptor determine the difference in desensitization, we found that the α1^ECD^/α3^TMD^β2 desensitized similarly to α3β2 and significantly different from α1β2, whereas α3^ECD^/α1^TMD^β2 displayed the second highest percentage residual current, close to the level exhibited by α1β2 (Fig 2). This points to that the molecular determinants underlying the difference between the residual currents characterizing α1β2 and α3β2 should be found in the transmembrane domain or the intracellular loops of the α-subunit. This is in agreement with previous reports of this region determining the desensitization of GABA_A_Rs [16]. The α1 and α3 TMDs are identical at the primary sequence level from TM1 through TM3, and thus the molecular difference between α1 and α3 TMDs resides in the intracellular loop between TM3 and TM4 (characterized by a very low degree of sequence homology between the two subunits) and in the TM4 and the short C-terminal extending from it (S1 Fig). A genetic mutation of a residue in this loop of the α1 GlyR subunit in hyperekplexia patients (A384P) has been found to increase the rate and extend of desensitization [22], and a switch of this loop between α1 and α3 GlyRs has been shown to transfer biophysical properties between the two channels [23]. It is also possible that the network of interactions between residues in the ECD and the TMD that underlies the translation of agonist binding into ion channel gating is responsible for the differences observed between α1β2 and α3β2. Finally, as the degree of residual current is determined relative to the peak current, the large residual currents we observed could in part reflect that we may not catch the full peak response evoked in the cells in our recordings, and consequently the residual current appears relatively larger. If the difference between the recorded and “true” peak response was more prominent for the α1β2 receptor than for the α3β2 receptor, it could even have skewed the observed relative difference in residual currents between the receptors.

## Conclusion

In the present study we were able to detect differences in biophysical properties between the α1β2 and α3β2 GABA_A_Rs using the whole cell patch clamp method. In agreement with previous studies performed using the excised patch clamp technique, the α1β2 receptor activated faster than the α3β2 receptor. While the two desensitized with similar kinetic profiles, a considerable difference in the fraction of desensitized receptors was observed with α3β2 desensitizing to a larger degree than α1β2. Our characterization of the biophysical properties exhibited by α1^ECD^/α3^TMD^β2 and α3^ECD^/α1^TMD^β2 chimeric receptors demonstrates that the major molecular determinants of these differences reside within the transmembrane and/or intracellular domains of the α-subunit.

## Supporting information

S1 Fig Alignment of the amino acid sequences of the transmembrane and intracellular domains of the human α1 and α3 GABA_A_R subunits. The sequences of α1 (black) and α3 (blue) are given with the transmembrane α-helices TM1-TM4 and the fusion point for the chimeric α1^ECD^/α3^TMD^ and α3^ECD^/α1^TMD^ subunits indicated. Conserved residues in the two segments are indicated with asterisks (*).
(DOCX)

S2 Fig Diazepam (1 μM)-mediated modulation of GABA (1 μM)-evoked currents in HEK293 cells expressing α1β2 γ 2S and α3β2 γ 2S GABA_A_Rs. Representative data (given as mean ± S.D) for modulation recorded at three different days following 3 different transfections.
(DOCX)

S1 Dataset (XLSX)

## References

[1] Olsen RW, Sieghart W. International Union of Pharmacology. LXX. Subtypes of γ-aminobutyric acid_A_ receptors: classification on the basis of subunit composition, pharmacology, and function. Update. Pharmacol Rev. 2008;60:243–60. Epub 2008/09/16. pr.108.00505 [pii] 10.1124/pr.108.00505 18790874 PMC2847512

[2] Chua HC, Chebib M. GABA_A_ Receptors and the Diversity in their Structure and Pharmacology. Adv Pharmacol. 2017;79:1–34. 10.1016/bs.apha.2017.03.003 . 28528665

[3] Rudolph U, Möhler H. GABA_A_ receptor subtypes: Therapeutic potential in Down syndrome, affective disorders, schizophrenia, and autism. Annu Rev Pharmacol Toxicol. 2014;54:483–507. 10.1146/annurev-pharmtox-011613-135947 24160694 PMC3997216

[4] Braat S, Kooy RF. The GABA_A_ Receptor as a Therapeutic Target for Neurodevelopmental Disorders. Neuron. 2015;86(5):1119–30. 10.1016/j.neuron.2015.03.042 . 26050032

[5] Stephens DN, King SL, Lambert JJ, Belelli D, Duka T. GABA_A_ receptor subtype involvement in addictive behaviour. Genes Brain Behav. 2017;16:149–84. 10.1111/gbb.12321 . 27539865

[6] Miller PS, Aricescu AR. Crystal structure of a human GABA_A_ receptor. Nature. 2014;512:270–5. 24909990 10.1038/nature13293 PMC4167603

[7] Laverty D, Desai R, Uchanski T, Masiulis S, Stec WJ, Malinauskas T, et al Cryo-EM structure of the human α1β3γ2 GABA_A_ receptor in a lipid bilayer. Nature. 2019;565(7740):516–20. 10.1038/s41586-018-0833-4 . 30602789 PMC6364807

[8] Zhu S, Noviello CM, Teng J, Walsh RM Jr., Kim JJ, Hibbs RE. Structure of a human synaptic GABAA receptor. Nature. 2018;559(7712):67–72. 10.1038/s41586-018-0255-3 29950725 PMC6220708

[9] Jones MV, Westbrook GL. Desensitized states prolong GABA_A_ channel responses to brief agonist pulses. Neuron. 1995;15(1):181–91. 10.1016/0896-6273(95)90075-6 . 7542462

[10] Jones MV, Westbrook GL. The impact of receptor desensitization on fast synaptic transmission. Trends Neurosci. 1996;19(3):96–101. 10.1016/s0166-2236(96)80037-3 . 9054063

[11] Miller PS, Smart TG. Binding, activation and modulation of Cys-loop receptors. Trends Pharmacol Sci. 2010;31(4):161–74. Epub 2010/01/26. S0165-6147(09)00211-9 [pii] 10.1016/j.tips.2009.12.005 . 20096941

[12] Haas KF, Macdonald RL. GABAA receptor subunit gamma2 and delta subtypes confer unique kinetic properties on recombinant GABAA receptor currents in mouse fibroblasts. J Physiol. 1999;514 (Pt 1):27–45. 10.1111/j.1469-7793.1999.027af.x 9831714 PMC2269054

[13] Chen X, Keramidas A, Lynch JW. Physiological and pharmacological properties of inhibitory postsynaptic currents mediated by α5β1γ2, α5β2γ2 and α5β3γ2 GABA_A_ receptors. Neuropharmacology. 2017;125:243–53. 10.1016/j.neuropharm.2017.07.027 . 28757051

[14] Barberis A, Mozrzymas JW, Ortinski PI, Vicini S. Desensitization and binding properties determine distinct alpha1beta2gamma2 and alpha3beta2gamma2 GABA(A) receptor-channel kinetic behavior. Eur J Neurosci. 2007;25(9):2726–40. 10.1111/j.1460-9568.2007.05530.x 17561840 PMC1950087

[15] Picton AJ, Fisher JL. Effect of the alpha subunit subtype on the macroscopic kinetic properties of recombinant GABA(A) receptors. Brain Res. 2007;1165:40–9. 10.1016/j.brainres.2007.06.050 17658489 PMC2084258

[16] Gielen M, Thomas P, Smart TG. The desensitization gate of inhibitory Cys-loop receptors. Nat Commun. 2015;6:6829 10.1038/ncomms7829 25891813 PMC4410641

[17] Jensen AA, Bergmann ML, Sander T, Balle T. Ginkgolide X is a potent antagonist of anionic Cys-loop receptors with a unique selectivity profile at glycine receptors. J Biol Chem. 2010;285:10141–53. 20106969 10.1074/jbc.M109.079319 PMC2843176

[18] Söderhielm PC, Balle T, Bak-Nyhus S, Zhang M, Hansen KM, Ahring PK, et al Probing the molecular basis for affinity/potency- and efficacy-based subtype-selectivity exhibited by benzodiazepine-site modulators at GABA_A_ receptors. Biochem Pharmacol. 2018;158:339–58. 10.1016/j.bcp.2018.08.019 . 30121248

[19] Hegazy NH, Breitinger HG, Breitinger U. Kavalactones from Kava (*Piper methysticum*) root extract as modulators of recombinant human glycine receptors. Biol Chem. 2019;400(9):1205–15. 10.1515/hsz-2019-0112 . 31141476

[20] Bianchi MT, Macdonald RL. Slow phases of GABA_A_ receptor desensitization: structural determinants and possible relevance for synaptic function. J Physiol. 2002;544(Pt 1):3–18. 10.1113/jphysiol.2002.020255 12356876 PMC2290568

[21] Karlsson U, Druzin M, Johansson S. Cl^-^ concentration changes and desensitization of GABA_A_ and glycine receptors. J Gen Physiol. 2011;138(6):609–26. 10.1085/jgp.201110674 22084415 PMC3226965

[22] Wang CH, Hernandez CC, Wu J, Zhou N, Hsu HY, Shen ML, et al A Missense Mutation A384P Associated with Human Hyperekplexia Reveals a Desensitization Site of Glycine Receptors. J Neurosci. 2018;38(11):2818–31. 10.1523/JNEUROSCI.0674-16.2018 29440552 PMC5852660

[23] Meiselbach H, Vogel N, Langlhofer G, et al Single expressed glycine receptor domains reconstitute functional ion channels without subunit-specific desensitization behavior. J Biol Chem. 2014;289(42):29135–47. 10.1074/jbc.M114.559138 25143388 PMC4200267

