# Supplementary material for: Comparison of biophysical properties of α1β2 and α3β2 GABAA receptors in whole-cell patch-clamp electrophysiological recordings
Source: PLoS One. 2020 Jun 1;15(6):e0234080. doi: 10.1371/journal.pone.0234080 (PMC7263626; doi:10.1371/journal.pone.0234080)
Supplement: S1 Fig — The sequences of α1 (black) and α3 (blue) are given with the transmembrane α-helices TM1-TM4 and the fusion point for the chimeric α1ECD/α3TMD and α3ECD/α1TMD subunits indicated. Conserved residues in the two segments are indicated with asterisks (*). (DOCX) [file pone.0234080.s001.docx]

**
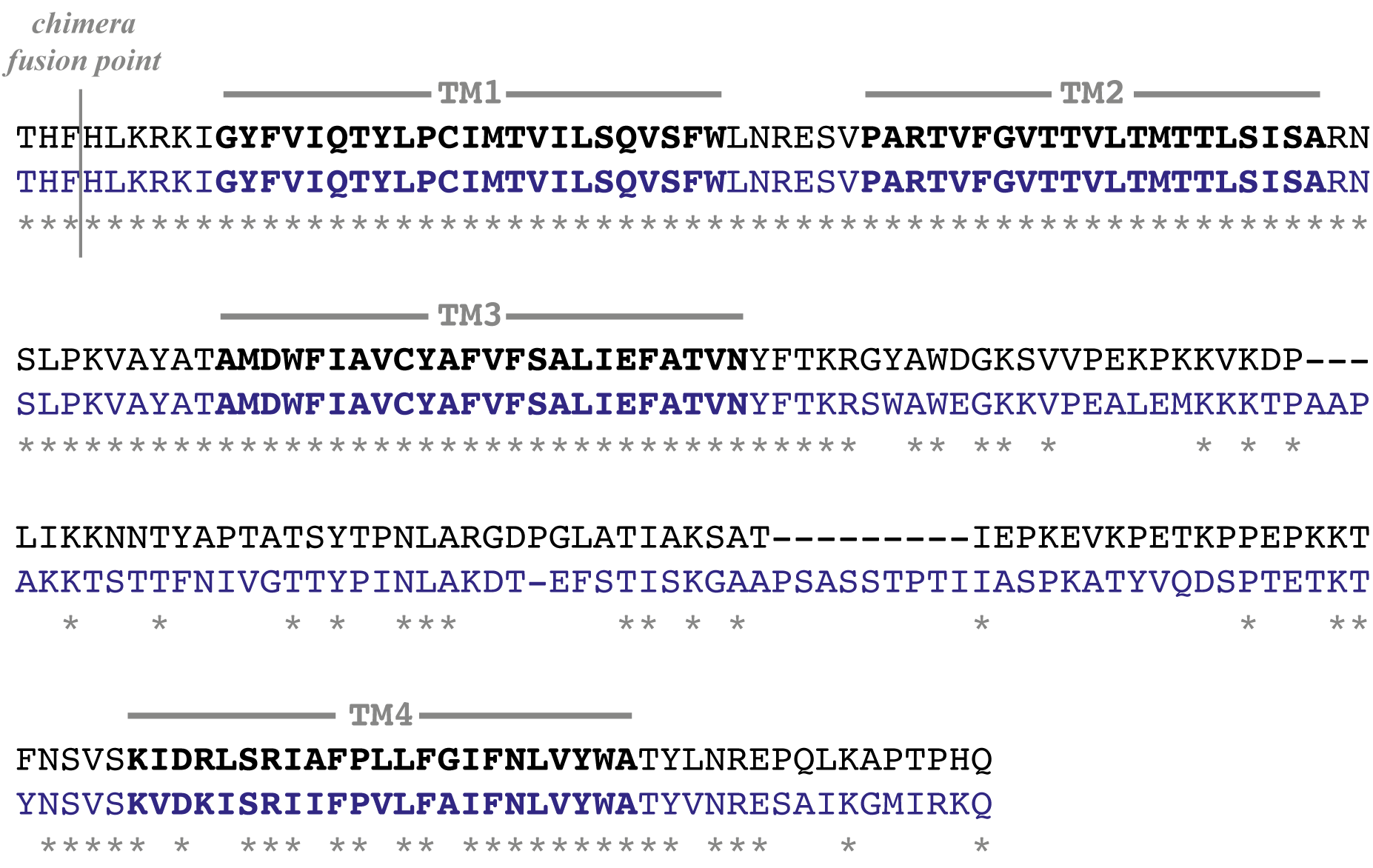
**

**SI Figure 1.** Alignment of the amino acid sequences of the transmembrane and intracellular domains of the human α1 and α3 GABA_A_R subunits. The sequences of α1 (black) and α3 (blue) are given with the transmembrane α-helices TM1-TM4 and the fusion point for the chimeric α1^ECD^/α3^TMD^ and α3^ECD^/α1^TMD^ subunits indicated. Conserved residues in the two segments are indicated with asterisks (*).
