# Supplementary material for: Comparison of biophysical properties of α1β2 and α3β2 GABAA receptors in whole-cell patch-clamp electrophysiological recordings
Source: PLoS One. 2020 Jun 1;15(6):e0234080. doi: 10.1371/journal.pone.0234080 (PMC7263626; doi:10.1371/journal.pone.0234080)
Supplement: S2 Fig — Representative data (given as mean ± S.D) for modulation recorded at three different days following 3 different transfections. (DOCX) [file pone.0234080.s002.docx]

**
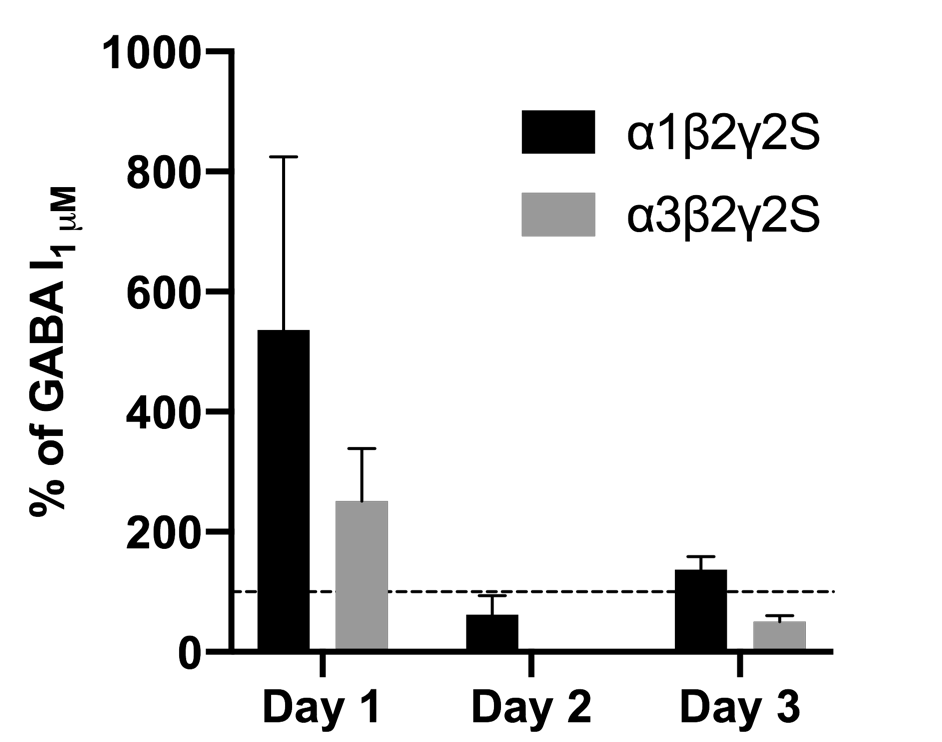
**

**SI Figure 2.** Diazepam (1 μM)-mediated modulation of GABA (1 μM)-evoked currents in HEK293 cells expressing α1β2γ2S and α3β2γ2S GABA_A_Rs. Representative data (given as mean ± S.D) for modulation recorded at three different days following 3 different transfections.
